# Quality of Life as reported by school children and their parents: a cross-sectional survey

**DOI:** 10.1186/1477-7525-6-34

**Published:** 2008-05-19

**Authors:** Thomas Jozefiak, Bo Larsson, Lars Wichstrøm, Fritz Mattejat, Ulrike Ravens-Sieberer

**Affiliations:** 1 The Norwegian University of Technology and Science (NTNU), Regional Centre of Child and Adolescent Mental Health MTFS N-7489, Dept. of Child and Adolescent Psychiatry St. Olav Hospital, 7000 Trondheim, Norway; 2 The Norwegian University of Technology and Science (NTNU) – Department of Psychology, N-7491 Trondheim, Norway; 3 Department of Child and Adolescent Psychiatry, Universitätsklinikum Gießen und Marburg, Hans-Sachs-Str. 6 35039 Marburg, Germany; 4 University of Bielefeld, School of Public Health – WHO Collaborating Center, Postfach 10 01 31 D-33501 Bielefeld, Germany; 5 Current Address : University Clinic Hamburg-Eppendorf, Center for Obstetrics and Pediatrics, Department of Psychosomatics in Children and Adolescents Building W 29 (Erikahaus)Martinistr. 52 D - 20246 Hamburg, Germany

## Abstract

**Background:**

Comprehensive evidence exists regarding the discrepancy between children's reports and parents' by proxy reports on emotional and behavioural problems. However, little is yet known about factors influencing the extent to which child self- and parent by proxy reports differ in respect of child Quality of Life (QoL). The aim of the study was to investigate the degree of discrepancy between child and parent by proxy reports as measured by two different QoL instruments.

**Methods:**

A representative Norwegian sample of 1997 school children aged 8–16 years, and their parents were studied using the Inventory of Life Quality (ILC) and the 'Kinder Lebensqualität Fragebogen' (KINDL). Child and parent reports were compared by t-test, and correlations were calculated by Pearson product moment coefficient. Psychometric aspects were examined in regard to both translated QoL instruments (internal consistency by Cronbach's alpha and test-retest reliability by intraclass correlation coefficients).

**Results:**

Parents evaluated the QoL of their children significantly more positively than did the children. Correlations between mother-child and father-child reports were significant (p < 0.01) and similar but low to moderate (r = 0.32; and r = 0.30, respectively, for the KINDL, and r = 0.30 and r = 0.26, respectively, for the ILC). Mother and father reports correlated moderately highly (r = 0.54 and r = 0.61 for the KINDL and ILC, respectively). No significant differences between correlations of mother-daughter/son and father-daughter/son pairs in regard to reported child QoL were observed on either of the two instruments.

**Conclusion:**

In the present general population sample, parents reported higher child QoL than did their children. Concordance between child and parent by proxy report was low to moderate. The level of agreement between mothers and fathers in regard to their child's QoL was moderate. No significant impact of parent and child gender in regard to agreement in ratings of child QoL was found. Both the child and parent versions of the Norwegian translations of the KINDL and ILC can be used in surveys of community populations, but in regard to the self-report of 9–10 years old children, only the KINDL total QoL scale or the ILC are recommended.

## Background

Epidemiological surveys of Quality of Life (QoL) are important and likely to provide valuable information for public health research as well as health service use. The use of generic instruments in both community and clinical populations enables comparisons between samples from these populations [1]. In contrast to research on QoL in adults, few studies of children and adolescents in the general population have been carried out using large representative samples [2-10] and which follows reliable QoL measures (we use "child " to denote children and adolescents in the paper).

To date, only a limited range of reliable and valid instruments have been developed for the assessment of QoL in children that fulfil the requisite criteria [11-16]. Such measures should reflect an acceptable definition of QoL and should not emphasize negative factors (ill-being). They should be multidimensional, and include physical, psychological and social well-being factors. QoL measure should also take account of the developmental stage of the child, be applicable to all children in a given culture, and be short and easy to use. Such measures should include child as well as parent proxy-report versions and have age-referenced general population norms. Further, a developmental framework is important when assessing pediatric QoL, because children's cognitive abilities, attitudes and subjective experience of their own well-being change across development [1].

In respect of the measurement of pediatric QoL, there is an ongoing debate in the literature concerning who is the most appropriate informant when there is a substantial discrepancy between child and parent reports of child health problems or child QoL [10,16-20]. It has been strongly emphasized that additional work is required to clarify the extent to which child and proxy ratings differ from each other in regard to QoL domain, health status, age and circumstances of the child [21,22].

In a recent study of QoL in healthy adolescents, low correlations between adolescent and parent reports were found, except for the school *domain *where correlations were moderate [23]. By contrast, agreement on child psychosocial-related QoL was higher between parents and *chronically sick *children as compared with parent reports and *healthy children *[21]. Further, degree of concordance between child and parent varied between *clinical groups *in studies of health-related QoL in children [24,25]. Higher agreement between parents and *children *(aged 7 to 11 years) compared to parents and *adolescents *has also been reported for a study of cancer patients [20].

Child and parent reports obtained in clinical and non-clinical (i.e. in a school population) settings are also likely to constitute different *circumstances *for the child. For example, it has been shown that parent-reported QoL scores in a clinical group of obese children were significantly lower than child reported scores on all but two domains [26]. In a preliminary analysis of a psychiatric outpatient sample, we found a similar tendency in that mother evaluations of their child's QoL were lower than child self-reports on most of the assessed domains [27]. In contrast, a study of a representative sample of 8–11 years old children from the general population concluded that children reported a significantly lower health-related QoL than did their parents on five out of seven of the assessed dimensions [10].

Although it has been recommended that the impact of *proxy gender in regard to gender of the child *should be investigated in QoL research [10], it appears that no such studies exist. In a recent Swedish controlled intervention study on parents' own QoL related to their asthmatic children, there were no major gender differences between mother and father ratings of QoL. However, mothers were more disturbed at night, and felt more helpless and frightened than fathers [28]. These findings indicate that mothers and fathers might be emotionally involved with their children in different ways, and that their reports of child QoL may be coloured by their own emotions [29].

In general, research evidence in regard to the influence of gender on child and parent agreement is contradictory. For example, in a study of links between parental adjustment and children's externalizing behaviour problems, sex composition of the parent-child dyad was found to be important in relation to parental adjustment patterns [30]. It has also been shown that mothers encourage children's illness behaviour more than fathers [31]. On the other hand, parents agree with each other on both higher and lower order personality traits in the child, and agreement between parents was not affected by child gender [32]. In a study of pre-pubertal children with mood disorders, the author did not find a significant relationship between child sex and parent-child differences scores for current or lifetime reports of mood disorder periods [33].

Further, in most child QoL research based on parent reports, the mother is usually the prime informant. If the generalization in the literature from mothers to "parents" is justified, it is important further to clarify whether important differences exist between mother and father ratings of child QoL.

For the purpose of the present study, we have defined "QoL" as "the subjective reported well-being in regard to the child's physical and mental health, self-esteem and perception of own activities (playing/having hobbies), perceived relationship to friends and family as well as to school."

The following two instruments were used: The Inventory of Life Quality (ILC) [34] and the 'Kinder Lebensqualität Fragebogen' (KINDL) [12,35]. These measures were developed in Germany for different purposes; the ILC as a brief screener in child psychiatry, and the KINDL for more extensive and broad assessment of QoL in children.

The primary aims of the study were to compare child and parent by proxy ratings of child QoL and to investigate factors influencing the degree of discrepancy in regard to these reports. We also evaluated internal consistency and test-retest reliability for the Norwegian translation of the child and parent versions of the KINDL and the ILC.

The following hypotheses were tested in respect of child and parent reports of QoL in a representative sample of Norwegian students aged 8–16 years:

(1) The magnitude of correlations between child and parent proxy report will be low to moderate. Because the study was conducted in the general population, we expected that parents would evaluate their children's QoL as higher than would the children themselves.

(2) Differences in correlations between mother-child and father-child reports of child QoL will be small. The impact of parent and child gender in regard to agreement in ratings of child QoL will be small, i.e. mother-daughter/son vs. father-daughter/son pairs.

## Methods

### Population and sample selection

The general population of students in the county of Sør-Trøndelag was stratified according to geography and grade: 4^th ^grade (age 9 or 10 years); 6^th ^grade (age 11 or 12 years); 8^th ^grade (age13 or 14 years) and 10^th ^grade (age 15 or 16 years). The national Norwegian database for primary education (GSI) was used to enumerate all pupils attending any of the targeted grades at all schools in the relevant region. Thus, 426 school grade cohorts were identified (a school grade cohort was defined by all pupils attending a specific grade at single school). After the exclusion of schools with a total of 50 pupils or less, and one international English-language school, 336 grade cohorts remained. Of these, 61 were randomly selected for the study. These comprised a total of 2,902 children attending 51 schools. Ninety-eight students had to be excluded because they either lacked sufficient competence in the Norwegian language (refugees, n = 51), and/or because they had an academic developmental level corresponding to more than two school grades below the respective grade (n = 47). Out of 2,804 students eligible for inclusion in the study, parents of 2,018 such students gave their active informed consent regarding their children's participation. However, 21 students did not meet appointments made by the local research coordinator. Thus, 1,997 students (990 girls and 1,007 boys) aged 8 – 16 years were finally included in the study, yielding a response rate of 71.2% (of 2804). For 1,777 of the 1,997 students, there was at least one caregiver who filled out the ILC, and for 1,743 students at least one caregiver filled out the KINDL. We included 1,188 and 1,169 complete mother-father pairs for the ILC and KINDL, respectively.

The number of 4^th ^grade students (8 – 10 year) was 505; 6^th ^grade students (10 – 12 years) 462; 8^th ^grade students (12 – 14 years) 492 and 10^th ^grade students (14 – 16 years), 538. The urban-to-rural resident ratio of children was 1:1.01 in the present sample, compared to 1.2:1 in the county, and the ratio of males to females was almost identical in the study sample (1.02:1) compared to the county (1.03:1).

### Assessment procedures

One teacher at each school was appointed as a project coordinator and given information about the research project and procedures for collecting the data. The coordinator informed the students about the project and also sent a standard information letter to their parents. The principal investigator (the first author) or a research assistant was present at each school when the students filled out the questionnaires. They stressed informant confidentiality, responded to questions, and read questions aloud for students with reading problems and all pupils in the 4^th ^grade. Completed questionnaires marked with an ID number were collected in closed envelopes by the researchers. A total of 104 students, who were not present the day of data collection, completed their questionnaires individually during the following week, under supervision of the local coordinator. To assess test-retest reliability, a subgroup of 143 students, aged 11–14 years (8^th ^grade students from one school in the sample, n = 88, and 6^th ^grade students from another school, n = 55, were retested after a two or a four-week period (response rate of 61%). The collection of data took place from September 2004 until June 2005, and October until November 2005.

### Measures

#### The Inventory for Assessing the Quality of Life (ILC)

This measure was developed in Germany by Mattejat and colleagues as a short and practical assessment tool for children and adolescents. It consists of 15 items [34] especially suited for use in clinical psychiatric settings. There are forms for children or adolescents, aged 7–18 years, and their parents. A Norwegian version of the generic 7-item ILC was used to assess various QoL areas over the past week. The ILC includes a global QoL score, and single-item subscales addressing school performance, family functioning, social integration, interest and hobbies, physical health and mental health. Each item is rated on a 1 – 5 Likert scale (1 *= *"Very good", 2 = "Rather good", 3 = "Mixed", 4 = "Rather bad" and 5 =" Very bad"). For children aged 7 – 11 years, the ILC is administered in a structured interview. Three types of scores can be calculated from the ILC. 1. The problem score (0 – 7) is computed by dichotomizing each of the seven items, such that ratings of 1 or 2 = 0 (no problem) and ratings of 3, 4 or 5 = 1 (present problem). 2. The QoL score LQ0-28 is calculated by multiplying the mean of the seven items by seven. 3. The QoL score LQ0-100 is the LQ0-28 divided by 28 and multiplied by 100.

In school populations, the German ILC has shown an internal consistency (Cronbach's α) of 0.63 (alpha = 0.76 for the parent version). Test-retest reliability was r = 0.72 for the LQ0-100 score (r = 0.80 for the parent version). The ILC has shown a moderate convergent validity with the KINDL [36]. German norms are available by gender and age, based on large scale studies of school samples (N = 9,364), parent ratings, and telephone interviews [3].

In the present study, the Norwegian translation of the ILC student report showed alpha values for the seven items in the four grades from 0.64 to 0.82 (see table 1). The alpha for the parent version of the ILC was 0.80. Two-week test-retest reliability for the Norwegian student report was high, and four-week test-retest reliability was moderate, for both ILC problem and ILC LQ28 score (se table 2). Student ratings on the ILC LQ0-100 and KINDL total 100 scales correlated moderately with each other (r = 0.69; p < 0.01; n = 1961).

**Table 1:** Internal consistency (Cronbachs alpha) coefficients for the KINDL and ILC. Student report by grade.

	**KINDL total scale**	**KINDL physical well-being**	**KINDL emotional well-being**	**KINDL Self-esteem**	**KINDL Family**	**KINDL Friends**	**KINDL School**	**ILC Item 1 – 7**
**Internal consistency**								
4^th ^grade (n = 500–503)	0.83	0.66	0.52	0.68	0.62	0.49	0.47	0.64
6^th ^grade (n = 449–458)	0.86	0.64	0.58	0.71	0.66	0.67	0.55	0.82
8^th ^grade (n = 483–492)	0.89	0.68	0.65	0.81	0.78	0.62	0.61	0.80
10^th ^grade (n = 531–537)	0.89	0.70	0.72	0.79	0.81	0.69	0.69	0.81

**Table 2:** Test-retest reliability (ICC) on the KINDL and ILC as reported by students by grade.

	**KINDL total scale**	**KINDL physical well-being**	**KINDL emotional well-being**	**KINDL Self-esteem**	**KINDL Family**	**KINDL Friends**	**KINDL School**	**ILC problem-score**	**ILC LQ28 score**
**Test-retest 2-week **(n = 28–31) 6^th ^grade	0.83***	0.52**	0.73***	0.64***	0.88***	0.78***	0.75***	0.91***	0.89***
**Test-retest 2-week **(n = 46–48) 8^th ^grade	0.90***	0.36**	0.67***	0.85***	0.87***	0.82***	0.84***	0.78***	0.84***
**Test-retest 2-week **(n= 75–79) **Total**	0.87***	0.43***	0.70***	0.77***	0.87***	0.81***	0.82***	0.83***	0.86***
**Test-retest 4-week **(n = 30–31) 6^th ^grade	0.54**	0.35*	0.37*	0.53**	0.59***	0.33*	0.54**	0.57***	0.70***
**Test-retest 4-week **(n = 35) 8^th ^grad0e	0.80***	0.13^n.s.^	0.46**	0.61***	0.72***	0.66***	0.80***	0.57***	0.72***
**Test-retest 4-week **(n = 65–66) **Total**	0.59***	0.26**	0.41***	0.59***	0.70***	0.47***	0.73***	0.59***	0.72***

*p < 0.05; **p < 0.01; ***p < 0.001.

*The KINDL *[12,35] has been developed for epidemiological use in healthy and clinical groups of children and adolescents aged 4 – 16 years. It encompasses separate generic forms for age groups 4 – 7, 8 – 12 and 13 – 16 years, and a proxy version for parents. The self-report for age 4 – 7 encompasses 12 items with three categorical answers. Only a total score is calculated. The other forms consist of 24 items equally distributed into the following six subscales: Physical well-being, emotional well-being, self-esteem, family, friends, and school. Each item addresses experiences over the past week and is rated on a 5-point scale (1 = "Never", 2 = "Seldom", 3 = "Sometimes", 4 = "Often" and 5 = "Always"). Mean scores are calculated for each of the six subscales and for the total scale and linearly transformed to a 0 – 100 scale.

For the German KINDL, internal consistency (Cronbach's α) has been reported at 0.70 and higher for the subscales and 0.80 for the total scale [12,35]. Correlations with comparable well-being scales have shown acceptable convergent validity, and a high correlation (r > 0.70) with subscales of the Child Health Questionnaire [37], as well a satisfactory discriminant validity [35].

The Norwegian translation of the adolescent version has been previously tested and Cronbach's alpha of 0.53 to 0.78 for the subscales, and 0.82 for the total scale have been reported [38]. In the present study, the internal consistency of the Norwegian KINDL increased with increasing age of the child with few exceptions (see table 1). The friends and school subscales showed the lowest alpha values in 4^th ^grade (0.49 and 0.47, respectively), while the family subscale showed the highest values in 10^th ^grade (0.81). For the KINDL total scale, alpha ranged from 0.83 in 4^th ^grade to 0.89 in 10^th ^grade. The parent versions of the KINDL subscales yielded alpha values from 0.67 to 0.80, and 0.89 for the KINDL total QoL scale. In regard to two-week test-retest reliability the student report for the total group (both 6^th ^and 8^th ^graders) showed high and significant ICC values on all scales and scores, except for the KINDL physical well-being subscale (ICC = 0.43) (se table 2). For the four-week retest, all ICC values decreased to a moderate level for the whole group, except for the KINDL physical well-being, emotional well-being and friends subscales, which produced low correlations (0.26, 0.41 and 0.47 respectively) (see table 2).

#### The translation process

Two independent forward, and one backward, translations of the ILC and the KINDL were completed. The forward translations were conducted by experienced Norwegian school teachers with a university degree in German. In addition, two bilingual children (a boy, aged 10 – 11 and a girl aged 13 – 14 years) also participated in the translations. The translators discussed semantic and conceptual discrepancies and finally developed a consensus-based forward translation. The ILC consensus forward translation was pilot tested in two girls (aged 9 and 13 years) and one boy (aged 10 years). The KINDL translation was also pilot tested in 11 school children (5 boys and 6 girls, aged 8 – 12 years) and seven parents. Children and parents reported their experience on a short questionnaire in regard to "How difficult it was to complete the questionnaire", "How items had been understood" and "How they liked the design of the instrument". It took 5 – 10 minutes for the children to complete the instruments and the majority were satisfied. The final Norwegian versions were translated back into German by a bilingual psychiatrist (ILC), and a professional translator (KINDL). The back-translations were approved by the developers. At that time, a Norwegian version of the adolescent KINDL form had already been established [38]. Efforts were therefore made to harmonize this version in the translation process for a common Norwegian KINDL version. The final Norwegian translations of the ILC and the KINDL are available on the internet [39,40].

*Socio-demographic information *on age and sex was obtained from the students and parents. In addition, parents provided information on their education.

### Ethics

The Norwegian Ethical Committee for Medical Research and the Norwegian Social Science Data Service approved the protocol.

### Statistics

Missing values were substituted by expectation maximization (EM) on the ILC. For the KINDL, we used mean substitution in descriptive statistics to facilitate comparison with the original German studies. Internal homogeneity was examined by Cronbach's α and test-retest stability by ICC. Correlations between continuous variables were calculated by Pearson product-moment coefficients. To compare correlations between different parent-child pairs, transformation into z-scores was used. Then, differences between z scores were calculated for the four parent child combinations (i.e. mother's minus daughter's z score, etc.) Further, means of these difference scores were compared by paired t-tests. Differences between two group means were analysed by independent t-test for continuous variables.

Differences in disagreement between informants on the ILC were analysed by the McNemar test. Effect sizes for between-group differences were calculated as recommended by Cohen [41]. Due to cluster-sampling of school units in the study, random-effects and between school variance were estimated by means of Mixed Linear Models [42]. An alpha level of p < 0.05 indicated statistical significance.

## Results

### Cluster effects

Due to our cluster sampling procedure, we first explored possible cluster effects. The results of an analysis of unconditional random effects showed that only 3.6% of the total variance of the ILC LQ0-28 scores and 6.5% of the total KINDL Total QoL scores could be explained by differences *between *the 61 school grade cohorts in the study. Further analysis of the six KINDL subscales showed low proportions for Physical well-being (2.6%), Emotional well-being (3.4%), Self-esteem (3.2%), Family well-being (6.3%) and Friends (3.2%). However, on the KINDL School subscale 13.9% of total variance was explained by differences between grade cohorts rather than by variation between pupils within each grade cohort.

Parental socio-economic level or school characteristics might explain differences between school grade cohorts. Therefore, we tested a two-level hierarchical model with parent education and size of school grade cohort at a cluster level, and parental education at the individual level, using the KINDL School subscale as the outcome variable. However, none of the covariates was significant. Because the QoL measures in the sample were only minimally influenced by differences *between *grade cohorts, all following analyses were conducted on an individual level.

### Child and parent report

#### Child report

**Figure 1:**
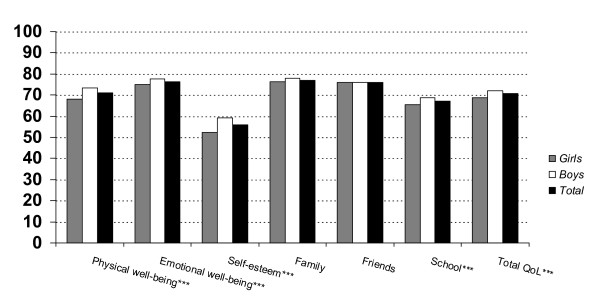
**Student report on the KINDL for girls, boys and the total sample (N = 1966^1^)**. ***Differences between sexes: p < 0.001 independent t-test (two-tailed). ^1 ^The difference in sample size to all included students in the study (N = 1997). reflects missing data on the KINDL.

QoL scores on KINDL total and subscales for boys, girls and total sample are shown in figure 1. Girls reported significantly (p < 0.001) lower QoL on the total scale and on four of the six subscales. However, effect sizes were low (1 – 3%). Prevalence rates of child reported problems on the seven ILC items were 23.3% for Physical health, 16.8% for Mental health, 23.3% for Perception of own activities (playing/having hobbies), 12.4% for Relationship to the family, 12.6% for Relationship to other children, 24.1% for Relationship to school, and 15.8% of the students reported problems with regard to their Global QoL.

#### Parent vs. child report

Pearson product-moment correlations between child and parent reports (at least one caregiver) on the KINDL and the ILC were significant but low for all subjects (r = 0.31 and 0.28, respectively) (see table 3). Further analysis related to school grade revealed that correlations were lower for the students in 4^th ^and 6^th ^grades (r = 0.23; p < 0.01; n = 887), as compared to those in 8^th ^and 10^th ^grades (r = 0.37; p < 0.01; n = 856) on the KINDL total QoL scale.

**Table 3:** Correlations^1 ^between mother, father and child reports on the KINDL total QoL and ILC LQ28 score^2^.

	Child	Daughter	Son	Mother	Father	At least one caregiver^3^
Child	-	-	-	0.32** N = 1180	0.30** N = 1175	0.31** n = 1743
Daughter	-	-	-	0.39** n = 589	0.34** n = 586	-
Son	-	-	-	0.26** n = 591	0.26** n = 589	-
Mother	0.30** n = 1197	0.31** n = 600	0.32** n = 597	-	0.54** N = 1169	-
Father	0.26** n = 1188	0.25** n = 594	0.29** n = 594	0.61** N = 1188	-	-
At least one caregiver^3^	0.28** n = 1777	-	-	-	-	-

^1 ^Pearsons product-moment correlations

^2 ^KINDL total QoL score correlations shown in **bold**; ILC LQ28 score correlations shown underlined.

^3^KINDL: Including 1657 mothers; ILC: Including 1689 mothers.

**p < 0.01.

**Figure 2:**
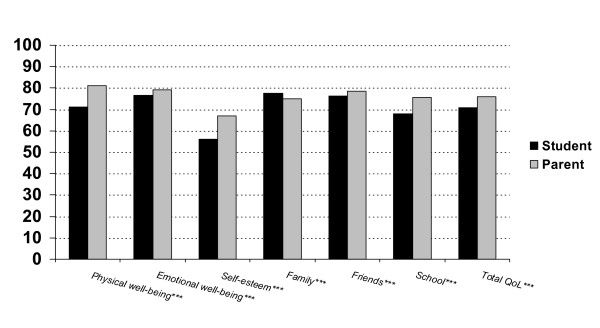
**Child and parent mean scores for different life domains on the KINDL (N = 1743)**. ***Mean differences between student and parent scores: p < 0.001, paired t-test (two-tailed).

**Figure 3:**
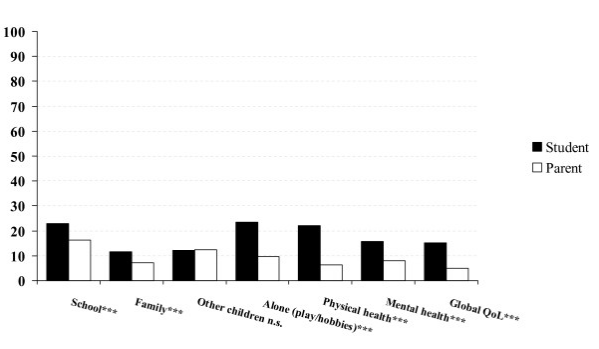
**The prevalence of reported problems in percentages on the ILC by 1777 child and parent pairs**. ***p < 0.001 χ^2^.

Figure 2 shows the ratings of 1,743 children and at least one parent (including 1,657 mothers) for different QoL domains and KINDL total QoL score. Except for the family domain, parental ratings of child QoL were significantly higher than were those of the children themselves. Effect sizes were 11% for physical well-being and self-esteem, 7% for the total QoL score and school, and 1% for emotional wellbeing, friends and family, representing small to medium effects. Figure 3 shows the prevalence of reported problems on the ILC as reported by all child and parent pairs on all seven domains. Significantly fewer parents than children reported problems for the child on almost all life domains.

Correlations between mother and father reports were significant and moderately high, both on the KINDL and the ILC (r = 0.54 and 0.61, respectively) (see table 3). Correlations between mother-child and father-child reports were low and almost identical on the KINDL, and similar on the ILC (range r = 0.26 to 0.32) (see table 3). Table 3 further shows that all computed correlations between mother and daughter, mother and son, father and daughter and father and son reports on the ILC and KINDL were significant, but small and similar (range r = 0.25 to 0.31, and 0.26 to 0.39, on the ILC and KINDL, respectively). However, no statistically significant differences *between *the means of the difference z-scores of the four parent-child pair combinations were found.

## Discussion

In this study of school-children aged 8–16 years and their parents, parents evaluated the QoL of their children significantly more positively than did the children themselves. Correlations between mother-child and father-child reports were similar and low, while the correlations between mother and father reports were moderately high. No significant differences between correlations of mother-daughter/son and father-daughter/son pairs in regard to reported child QoL were observed on either of the two instruments. The Norwegian versions of ILC and KINDL showed an overall satisfactory internal consistency and test-retest reliability on both the child and parental versions, except for the KINDL subscales for children aged 9–10 years.

Overall, the quality of our data was satisfactory with very low rates of missing values. A detailed analysis showed that the present selected school sample was representative for the general population of the county in regard to male:female and urban:rural ratios as well as age range. Because the QoL measures in the sample were only minimally influenced by differences *between *grade cohorts, statistical analyses could be conducted on an individual level.

### Child and parent report

With regard to the child report, observed sex differences on the KINDL were significant in that girls reported a lower QoL than boys, but all differences had a low effect size. Our results were consistent with outcomes of previous research in that girls reported a lower QoL than boys [2,4,43]. Further, it is notable that the highest proportion of problems reported on the ILC was in the school domain. On the other hand, the children reported lowest problems in relation to their families.

According to our first hypothesis, correlations between child and parent reports of child total QoL in the present study were low to moderate for both the KINDL and ILC measures. These results are also consistent with previous research [i.e. [10,16-21,24,25]]. We expected a pattern of parent reports, where parents would report a higher child QoL than the children themselves because our sample was based on a general population and not a clinical sample. Our results confirmed the hypothesis with parental ratings of child QoL being significantly higher than those of the children. However, the associated effect sizes varied from low to moderate to high for the different subscales. With regard to the child's ratings of physical well-being, self-esteem, school and total QoL scores, the child-parent divergence was moderate to high. The prevalence of reported problems on the ILC mirrored the hypothesized trend in that children reported more problems on most of the domains than did their parents' in regard to child QoL, thereby supporting our hypothesis. Previous research has shown the opposite trend among children and adolescents with psychiatric problems, in that parents rated child QoL significantly lower than did the children [27]. Parental evaluations of children referred to psychiatric services might be influenced by the parents' anxieties or worries. Almost 90% of the patient's mothers reported that they were stressed due to their child's disorder, while only about 50% of the patients did [27]. In a clinical study of obese children parental ratings showed a similar trend in that parent report of child QoL was significantly lower than those of the children in social and emotional QoL domains [26]. However, this trend was not observed in school-, and physical domains. In the present study, these two domains contributed to high divergence and reports of higher child QoL by the parents as compared to child report. Further, rates of concordance between child and caregiver varied between clinical groups in line with findings recently reported by Wilson-Genderson et al. [24].

Another potential factor that may impact on the degree of child-parent discrepancy is the child's age. For example, Chang and Yeh [20] reported greater agreement between younger children (up to 12 years) vs. older children in both self and parental ratings of QoL [20], which is in contrast to the results of the present study. We also observed that correlations between child (8 – 12 years) and parent ratings were lower than between adolescents and parents. This discrepancy in findings may be due to differences in sample characteristics, in that the Chang and Yeh study included children with cancer, while our results were obtained in a general student population. Further research is needed to clarify whether the child's age has a systematic influence on the discrepancy between child and parent reports of QoL.

Psychometric properties of QoL measures also have to be considered in regard to child's age. The present study showed that ratings of younger children generally yielded lower internal consistency than older ones, with few exceptions. Maturation of the child's cognitive abilities [1,17] might be an explanation of the observed trend. The formulation of certain items might have lead to a larger degree of variability in the understanding of their meaning by younger children than by older ones. Thus, the observed low internal consistency on the KINDL Emotional well-being-, Friends-, and School – subscales for children in 4^th ^grade could represent serious obstacles with respect to the interpretation of results. Therefore, in accordance with the original author [4], we will recommend the use of the KINDL total QoL scale for this age-group, which showed a satisfactory internal consistency. The ILC consisting of 7 items, could also be a good alternative to a longer instrument, where the main purpose would be to obtain a reliable overall child report; for example, in a busy clinical context with disordered children who experience problems filling out longer instruments. The ILC can also be used in broad-scaled epidemiological surveys, where instruments cannot be too long but must still provide reliable scores. Where it is not possible to provide self-reports on child QoL [16], either due to the young age of the child or to other circumstances, both the Norwegian ILC and the KINDL parent version may be used given their satisfactory internal consistency. However, one must bear in mind that the correlations between child and parent reports of child total QoL are only low to moderate. Consequently, parent evaluation of child QoL cannot represent a real substitute for the child's own perspective.

Our second hypothesis was that differences in correlations between mother-child and father-child reports of child QoL would be small. This was supported in that the size of father vs. child, and mother vs. child correlations were almost identical on the KINDL and similar on the ILC. We further hypothesized that the impact of parent and child gender in relation to agreement in ratings of child QoL would be small. This was supported in that we did not observe significant differences between correlations of mother-daughter/son and father-daughter/son pairs. Our findings are notable given that father participation in previous studies of QoL in children was much lower than in the present study. Therefore, our results could support (and justify) the generalization from "mothers" to "parents" that is often made in QoL research reports. On the other hand, the present study was conducted in a Scandinavian country, where equal status of the sexes is well established as a cultural ideal. As Hederos et al. in Sweden have pointed out, most of the mothers work outside their homes. Hence the fathers have to engage more in their children's care, which is also encouraged by the authorities through shared paid leave in connection with the birth of the child [28]. The situation is very similar in Norway, and our findings should not be generalised to countries with a different gender role structure. The possible impact of sex differences in parent reports on the degree of discrepancy between child and parent report needs still to be investigated.

Although sex differences in parent and child pairs were nonsignificant in the present study, we found that mother's and father's QoL by proxy reports correlated only moderately. This may be interpreted as an indication of substantial disagreement in their views on QoL in the child.

Finally, we certainly agree with Eiser & Morse [21] about the importance of relating observed parent and child disagreement to the circumstances of the child. Our findings, together with recent research reports on this matter, suggest that an evaluation of the child's circumstances should always include dimensions such as "healthy vs. ill", "clinical or non-clinical setting", "group of disease", "age of the child" and "the source of the by proxy informant and his/hers personal characteristics". Rather than considering parent-child disagreement only as a potential bias of the instrument in question, disagreement is also likely to reflect the different perspectives of informants in various contexts [16].

### Limitations of the study

About 10% of parents whose children participated in the study did not fill out the QoL questionnaires. The group of children with at least one parent filling out the questionnaire reported significantly lower total QoL levels on the KINDL, but did not differ from other children on the physical health, self-esteem and friends KINDL subscales. It is likely that these differences in response rates represent parental bias in terms of slight overestimates of QoL levels in their children.

## Conclusion

In the present general population sample, parents reported higher child QoL than did their children. Concordance between child and parent by proxy report was low to moderate, and mothers and fathers agreed moderately in regard to their child's QoL. Further, no significant impact of parent and child gender in regard to agreement in ratings of child QoL was found. Both the child and parent by proxy versions of the Norwegian translations of the KINDL and ILC can be used in surveys of community populations. However, in regard to reports of 9–10 year old children, only the KINDL total QoL scale or the ILC are recommended. Rather than considering parent-child disagreement only as a potential bias of the instrument in question, disagreement is also likely to reflect different perspectives of informants in various contexts.

## Competing interests

The authors declare that they have no competing interests.

## Authors' contributions

TJ contributed to the study design, data collection, statistical analysis, interpretation of data and the drafting of the paper. BL contributed to the study design, statistical analysis, interpretation of data and the revising of the manuscript. LW made contribution to the study design, statistical analysis, interpretation of data and the revision of the manuscript. FM is the original author of the ILC, and made a contribution to the translation process of the Norwegian ILC, statistical analysis and the revision of the manuscript. URS is the original author of the KINDL, and made a contribution to the translation process of the Norwegian KINDL, statistical analysis and the revision of the manuscript. All authors read and approved the final manuscript.
